# A Case Study for Effects of Operational Taxonomic Units from Intracellular Endoparasites and Ciliates on the Eukaryotic Phylogeny: Phylogenetic Position of the Haptophyta in Analyses of Multiple Slowly Evolving Genes

**DOI:** 10.1371/journal.pone.0050827

**Published:** 2012-11-30

**Authors:** Hisayoshi Nozaki, Yi Yang, Shinichiro Maruyama, Toshinobu Suzaki

**Affiliations:** 1 Department of Biological Sciences, Graduate School of Science, University of Tokyo, Tokyo, Japan; 2 Department of Biochemistry and Molecular Biology, Dalhousie University, Halifax, Nova Scotia, Canada; 3 Department of Biology, Graduate School of Science, Kobe University, Kobe, Japan; East Carolina University, United States of America

## Abstract

Recent multigene phylogenetic analyses have contributed much to our understanding of eukaryotic phylogeny. However, the phylogenetic positions of various lineages within the eukaryotes have remained unresolved or in conflict between different phylogenetic studies. These phylogenetic ambiguities might have resulted from mixtures or integration from various factors including limited taxon sampling, missing data in the alignment, saturations of rapidly evolving genes, mixed analyses of short- and long-branched operational taxonomic units (OTUs), intracellular endoparasite and ciliate OTUs with unusual substitution etc. In order to evaluate the effects from intracellular endoparasite and ciliate OTUs co-analyzed on the eukaryotic phylogeny and simplify the results, we here used two different sets of data matrices of multiple slowly evolving genes with small amounts of missing data and examined the phylogenetic position of the secondary photosynthetic chromalveolates Haptophyta, one of the most abundant groups of oceanic phytoplankton and significant primary producers. In both sets, a robust sister relationship between Haptophyta and SAR (stramenopiles, alveolates, rhizarians, or SA [stramenopiles and alveolates]) was resolved when intracellular endoparasite/ciliate OTUs were excluded, but not in their presence. Based on comparisons of character optimizations on a fixed tree (with a clade composed of haptophytes and SAR or SA), disruption of the monophyly between haptophytes and SAR (or SA) in the presence of intracellular endoparasite/ciliate OTUs can be considered to be a result of multiple evolutionary reversals of character positions that supported the synapomorphy of the haptophyte and SAR (or SA) clade in the absence of intracellular endoparasite/ciliate OTUs.

## Introduction

Reconstruction of the true phylogenetic history of organisms has been one of the most important biological issues since Charles Darwin proposed his evolutionary theory [1]. Advances in molecular phylogenetic analyses have allowed biologists to hypothesize concrete evolutionary histories of given organisms. However, phylogenetic analyses based on limited sequence data from a single gene often result in ambiguous branches. Recently, problems with such low statistical support values in phylogenetic resolution have apparently been resolved using concatenated sequences of multiple genes in various lineages of organisms [2]–[3], [4]–[5], [6]–[7], [8].

In the macrophylogeny of eukaryotic organisms, recent multigene phylogenetic studies have resolved deep branches of eukaryotes with apparently high support values. However, phylogenetic positions of various eukaryotic lineages have remained unresolved or in conflict among differing phylogenetic studies. For example, the phylogenetic position of the secondary photosynthetic eukaryotes haptophytes [9]–[10], one of the most abundant groups of oceanic phytoplankton and significant primary producers [11]–[12], is inconsistent in recent multigene phylogenetic studies. On the basis of more than 100 nuclear protein sequences, Patron et al. [13] resolved the monophyly of the haptophytes and cryptophytes (HC clade) and the HC clade was closely related to the Archaeplastida. In contrast, Burki et al. [14] showed nonmonophyly of the HC clade and a close relationship between the haptophytes and SAR (stramenopiles, alveolates, rhizarians), based on more than 200 nuclear protein sequences. These phylogenetic ambiguities could have resulted from mixtures of various factors including limited taxon samplings [15], missing data in the alignment [16], saturation of substitutions in rapidly evolving genes [17], mixed analyses of short- and long-branched operational taxonomic units (OTUs) [18], and intracellular endoparasite and ciliate OTUs with unusual substitutions.

Since the 1980s, sequence data from intracellular endoparasites, such as the amitochondrial eukaryotes, Kinetoplastida, and apicomplexans, have been available [19]–[20], primarily because these organisms are medically important. However, intracellular endoparasites, especially amitochondrial eukaryotes, exhibit unusually high numbers of gene substitutions and this can cause artifacts in resolving phylogeny [19]–[21]. Gribaldo and Philippe [20] pointed out the unnatural phylogenetic positions of endoparasites, including amitochondrial excavates and microsporidia, in previous phylogenetic analyses. Nozaki et al. [22] excluded amitochondrial excavates and microsporidians from their macrophylogeny of eukaryotes. Phillipe et al. [23] also performed a phylogenetic study of all eukaryotes without amitochondrial excavates and microsporidians. Furthermore, because ciliates have atypical patterns of gene transcription and translation [24]–[25], Nozaki et al. [26]–[27] excluded ciliates and intracellular endoparasites of Kinetoplastida (e.g., *Trypanosoma*, *Leishmania*) from their eukaryotic macrophylogenies. However, very recent multigene phylogenetic analyses of eukaryotes [28]–[29] have included amitochondrial excavates and ciliates. Based on taxon-rich analyses of 16 relatively slowly evolving nuclear genes, Parfrey et al. [29] indicated a close relationship between Archaeplastida and the HC clade, though with weak statistical support values.

This study was conducted to evaluate the effects of the presence or absence of intracellular endoparasite and ciliate OTUs on the phylogenetic positions of other eukaryotic lineages. In order to simplify the results, we used two different sets of data matrices of multiple slowly evolving genes with small amounts of missing data and examined the effects of intracellular endoparasite and ciliate OTUs on the phylogenetic position of the Haptophyta.

## Materials and Methods

We selected two different sets of multiple, slowly evolving nuclear genes that have been used for the macrophylogeny of eukaryotes [27]–[29], because use of these data sets helps to reduce artificial resolution of deep phylogeny due to rapidly evolving genes [17] and large amount of missing data in the alignment [16]. One of the two sets (“6,048 aa”) included a data matrix that was updated and modified from the 6,048 amino acids from 35 OTUs used previously (6,048×35 matrix [27]). The modifications include the exclusion of the green alga *Dunaliella* (lacking EF-1 alpha) and the very short-branched OTUs of glaucophytes (avoiding co-analyses of short- and long-branched OTUs), the addition of two free-living stramenopiles (*Aureococcus* and *Ectocarpus*) and three free-living excavates (the jakobid *Seculamonas* and two euglenoids, *Euglena* and *Peranema*), and the use of the *Coccomyxa* EF-1alpha sequence in place of the *Chlamydomonas* OTU (Table S1). Additional sequence data were extracted from databases of the National Center for Biotechnology Information (NCBI; http://www.ncbi.nlm.nih.gov/) (*Ectocarpus*, *Seculamonas*, and *Euglena*), the DOE Joint Genome Institute (JGI; http://www.jgi.doe.gov/) (*Aureococcus*), and our unpublished expressed sequence tag (EST) database (*Peranema*) [30]. For some short EST sequences from *Peranema*, colony PCR products of cDNA clones were sequenced directly, using an ABI PRISM 3100 Genetic Analyzer (Applied Biosystems, Foster City, CA, USA) with the BigDye Terminator Cycle Sequencing Ready Reaction Kit (v. 3.1; Applied Biosystems). The constructed 6,048 aa matrix contained 37 OTUs and 5.9% missing data (Submission ID: 13511, available from TreeBASE at http://www.treebase.org/treebase-web/home.html). To examine the effects of intracellular endoparasites on phylogenetic resolution, three apicomplexan OTUs were excluded for comparison.

The other analyzed set (M 10∶16) was modified from “10∶16″ of Parfrey et al. [29], which includes all OTUs that have at least 10 of the 16 genes, with a total of 88 OTUs, representing 26 lineages and containing 17% missing data. We also excluded two OTUs with ca. 50% missing data: *Brevitata* (a single OTU with an ambiguous phylogenetic position) and *Reticulomyxa* (very long-branched OTU [29]). Comparisons of phylogenetic results were performed between the M 10∶16 matrices retaining and excluding intracellular endoparasites (apicomplexans, endoparasite kinetoplastids, microsporidians, and endoparasite excavates)/ciliates (86 and 67 OTUs, respectively).

For both sets of data matrices, maximum parsimony (MP) trees were constructed using a heuristic search with the step-wise addition of 10 random replications (with the tree bisection-reconnection branch-swapping algorithm) using PAUP 4.0b10 [31]. The bootstrap values (BV) [32] for the MP analyses were calculated for 1,000 replications of the step-wise addition of 10 random replications.

The 6,048 aa matrices, composed only of amino acids, were analyzed by two maximum likelihood (ML) methods: PhyML 2.4.4 [33] and RAxML ver. 7.0.4 [34]. When the phylogenetic analyses were conducted with PhyML and RAxML for the 6,048 matrix, the Whelan and Goldman (WAG) model [35] was used, and a discrete model of site heterogeneity and invariable sites was assumed. Shape parameters used for ML analyses were estimated using PhyML and RAxML, and the number of rate categories for the distribution was set to four (a 4G model). The robustness of the lineages of the ML tree generated by PhyML and RAxML was tested by bootstrap analyses, based on 1,000 replications with PhyML and 1,000 rapid bootstrap inferences by RAxML. Because M 10∶16 contained both amino acid and nucleotide sequences [29], the ML analysis was performed only with RAxML ver. 7.2.6 [34]. For RAxML analysis of M 10∶16, WAG+4G and GTR+4G models were used for amino acid and nucleotide data, respectively, with 1,000 bootstrap replicates.

To examine characters that directly affected the sister relationship between haptophytes and SAR (SA) when intracellular endoparasite/ciliate OTUs were included, synapomorphic amino acid characteristics for the clade composed of the haptophytes and SAR or SA were determined based on character optimization by MacClade 4.08a [36] (using the option “ambiguous changes only”), using fixed trees showing monophyly between the haptophytes and SAR (or SA) (Figures S1, S2, S3).

## Results

### 6,048 aa

**Figure 1:**
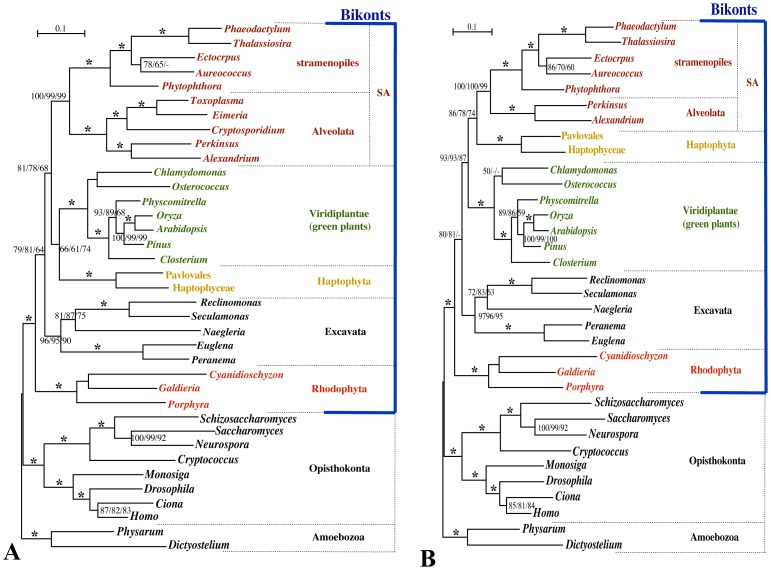
Eukaryotic phylogeny based on nuclear-encoded protein sequences using 6,048 aa (modified from the data matrix of Nozaki et al.[**27**]), including (A) and excluding (B) the intracellular endoparasites apicomplexans. The analysis is based on the concatenated dataset of slowly evolving nuclear proteins (21 proteins; 6,048 amino acid positions). The tree was determined using RAxML with the WAG+I+4G model. Numbers on the left, middle, or right side at the branches represent BV (≥50%) obtained using 1,000 replicates with the RAxML, PhyML (WAG+I+4G), or MP analysis, respectively. Asterisks at the branches indicate 100% BV by all three methods.

Based on analyses of 37 OTUs (including apicomplexans), red algae and excavates were recovered by basal lineages whereas SA, green plants, and haptophytes formed a monophyletic group with 78–81% and 68% BV in the ML and MP methods, respectively, within the bikonts or Super Plant Kingdom [27] (Figure 1A). Green plants and haptophytes formed a moderate monophyletic group (with 61–74% BV in MP and ML analyses), and this monophyletic group was a sister to SA (including three apicomplexan OTUs; Figure 1A). Nozaki et al. [27] resolved weak sister relationship between the haptophytes and SA in the 6,048 aa data matrix that included apicomplexans. The difference in the phylogenetic position of the haptophytes between the present study (Figure 1A) and Nozaki et al. [29] may have arisen from the difference in OTUs. The present study used increased OTUs from free-living excavates and excluded the short-branched glaucophytes (see Materials and Methods).

When the three apicomplexan OTUs were excluded, the haptophytes and SA formed a monophyletic group (SA-H clade) with moderate-to-robust statistical support (74–86% BV; Figure 1B). Additionally, the BV supporting the large monophyletic group composed of SA, green plants, and haptophytes increased compared with those using the 6,048 aa data matrix with the three apicomplexans included (79–81% vs. 87–93%; Figure 1B). Other phylogenetic results were not markedly different between the analyses including and excluding the apicomplexans.

Based on searches for unambiguous character changes in the fixed trees with MacClade, the number of amino acid positions where characters changed on the branch bearing SA and haptophytes (the SA-H branch) was 45 or 37 when excluding or including apicomplexan OTUs, respectively. These positions possibly represented derived amino acid characters that evolved at the SA-H branch, largely corresponding to the synapomorphic characters for the SA-H clade. Among the 45 positions, 13 were not found in the 37 positions determined in the fixed tree that included apicomplexan OTUs. In 11 of the 13 positions, the ancestral amino acids determined just below the SA-H branch (arrows, Figure S1) did not change between the optimizations excluding and including the apicomplexan OTUs. Such “ancestral” amino acids were determined for apicomplexans within SA in 10 of the 11 positions in the fixed tree (isoleucine, Figure S1B).

### M 10∶16

**Figure 2:**
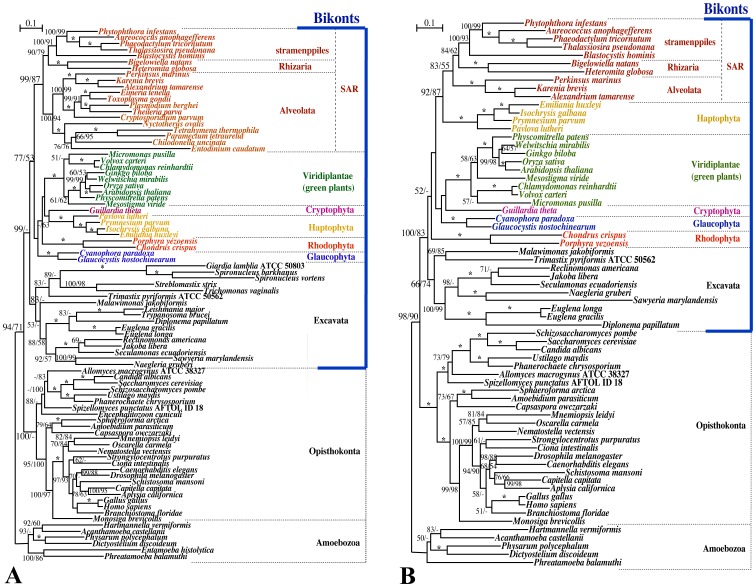
Eukaryotic phylogeny based on nuclear-encoded protein sequences plus nucleotide sequences of 18S rRNA genes using M 10:16 (modified from the data matrix by Parfrey et al.[**29**]), including (A) and excluding (B) intracellular endoparasite/ciliate OTUs. The analysis is based on the concatenated dataset of slowly evolving nuclear proteins (15 proteins; 5710 amino acid positions) and 18S rRNA genes (868 nucleotide positions). The tree was prepared using RAxML with the WAG+4G model (for amino acid positions) and the GTR+4G model (for nucleotide positions). Numbers at the left or right side at the branches represent BV (≥50%) obtained using 1,000 replicates with the RAxML or MP analysis, respectively. Asterisks at the branches indicate 100% BV by all three methods.

Phylogenetic results with the M 10∶16 data matrix of 86 OTUs were essentially the same as those reported by Parfrey et al. [29] except for weak resolution of the association between Archaeplastida and the HC clade in the present study (Figure 2A). Within the bikonts, SAR, Archaeplastida, cryptophytes, and haptophytes formed a large monophyletic group, with 53–77% BV. SAR represented a robust clade, with 87–99% BV. However, phylogenetic relationships between the three groups of Archaeplastida, cryptophytes, and haptophytes were not well resolved (Figure 2A).

When the OTUs from intracellular endoparasites and ciliates were removed (67 OTUs), the HC clade was not resolved; however, robust monophyly between SAR and the haptophytes was supported with 87–92% BV in MP and ML analyses. Other phylogenetic relationships were not as robustly resolved within the bikonts as those including intracellular endoparasite and ciliate OTUs (Figure 2B).

Based on searches for unambiguous character changes in the fixed trees with MacClade, the number of positions where characters changed on the branch bearing SAR and haptophytes (the SAR-H branch) was 55 or 47 when excluding or including, respectively, intracellular endoparasite/ciliate OTUs. Among the 55 positions, 28 were not found in the 47 positions in the fixed tree including endoparasite/ciliate OTUs. In 18 of the 28 positions, the ancestral characters suggested at the base of the SAR-H branch differed between the optimizations excluding and including the intracellular endoparasite/ciliate OTUs (arrows, Figure S2). Among the other 10 positions, for which characters determined at the base of the SA-H branch were unchanged between the two optimizations (arrows, Figure S3), seven showed ancestral characters in the branches bearing apicomplexans and/or ciliates within SAR in the fixed tree that included intracellular endoparasite/ciliate OTUs (alanine, Figure S3).

## Discussion

Based on the two different types of data matrix sets that were modified from Nozaki et al. [27] and Parfrey et al. [29], we obtained similar results for the phylogenetic position of the haptophytes in relation to the presence or absence of OTUs from intracellular endoparasites/ciliates. When co-analyzed with intracellular endoparasite/ciliate OTUs, the haptophytes were not sisters to SA or SAR and were weakly or moderately associated with Archaeplastida as in Parfrey et al. [29]. When excluding the intracellular endoparasite and ciliate OTUs, however, the haptophytes represented a sister group to SA or SAR, with 76% or more BV (Figures 1, 2). Patron et al. [13] showed robust nonmonophyly of SA when co-analyzed with endoparasite excavates (*Trypanosoma*, *Giardia*, *Trichomonas*), whereas they showed robust monophyly of SA when excluding the endoparasite excavates, as in other multigene phylogenies [27]. Thus, the effects of intracellular endoparasite/ciliate OTUs on other OTUs seem considerable and likely mislead the phylogenetic inferences about other OTUs. These effects are often recognized collectively as long-branch attraction [37].

Multigene phylogenetic analyses often include rapidly evolving genes that may be expected to cause artefactual resolution of phylogeny in deep branching [17]. Thus, Nozaki et al. [26] analyzed only slowly evolving nuclear genes and resolved robust nonmonophyly of the Archaeplastida. Furthermore, by analyzing such slowly evolving nuclear genes, Nozaki et al. [27] suggested a close relationship between the haptophytes and SA because the haptophytes have short branches and this relationship became robust when long-branched OTUs were removed. Their conclusion was based on Nei’s work [18], which suggested that co-analyses of short- and long-branched OTUs might fail to recover the true topology. Archaeplastida, haptophytes, and cryptophytes generally exhibit short branches, whereas SAR display long branches, especially ciliates and intracellular endoparasites, in multiple nuclear protein phylogenies [13]–[27], [28]. Thus, the effects of the presence or absence of ciliate and intracellular endoparasite OTUs on the phylogenetic position of the haptophytes resolved in the present phylogenetic analyses (Figures 1 and 2) can also be explained by whether short- and long-branched OTUs are co-analyzed.

In one of the data matrices used in the present study (6,048 aa), the intracellular endoparasite/ciliate OTUs included only three apicomplexans; however the exclusion of these three OTUs resulted in the drastic change in the phylogenetic position of the haptophytes (Figure 1). Character optimization on fixed trees constraining a haptophyte/SA clade indicated that including intracellular endoparasites (only apicomplexans in this case) and ciliates has a clear effect on how potentially synapomorphic characters are interpreted. Using the 6,048 aa data matrix, we identified 13 positions that suggested unique character change within the SA-H branch when apicomplexans were excluded, but not with apicomplexans present. The majority (10/13) of these positions appeared as synapomorphies or derived characters on the branch supporting the SA-H clade when apicomplexans were excluded. When apicomplexans were present within the SA clade, however, they exhibited “ancestral” amino acid characters, resulting in the loss of apparent synapomorphies that provided strong support for the SA-H clade (Figure S1). Ito et al. [38] suggested that rates of amino acid substitution in endocellular symbionts are elevated due to increased mutation rates. In our analyses this appears to result in reversals or multiple substitutions to more ancestral amino acid characters in the apicomplexan lineage (Figure S1). This, in turn, weakens tree-building signal from synapomorphic characters, which can explain the nonmonophyly of the SA-H clade in the phylogenetic analyses that include the three apicomplexans (Figure S1).

Similar possible reversals in evolution of characters in apicomplexans and ciliates could be considered in nine positions in the other data matrix set (M 10∶16, Figure S3). However, M 10∶16 exhibited changes in ancestral characters below the SAR-H branch in 18 of 28 positions that showed loss of unambiguous character evolution on the branch (possibly corresponding to loss of synapomorphies for the SAR-H clade) in the fixed tree when endoparasite/ciliate OTUs were included (Figure S2). Thus, the effects from intracellular endoparasite OTUs outside the SAR (endoparasite excavates, amitochondrial amoeba, and microsporidians) on the phylogenetic results seemed considerable in M 10∶16.

## Supporting Information

Figure S1 **Character optimization of one of the amino acid positions that directly affected the sister relationship between haptophytes (H) and the clade composed of stramenopiles and alveolates (SA), in relation to the absence (A) or presence (B) of the intracellular endoparasite apicomplexan OTUs (AP) in the 6,048 aa data matrix set.** Optimization was conducted using MacClade 4.08a in the fixed tree showing the clade composed of haptophytes and SA. The arrow indicates the ancestral character determined below the SA-H branch (bearing the clade composed of haptophytes and SA). Note that the derived character resolved at the SA-H branch (A) disappeared in the lower tree (B).
(TIF)

Figure S2 **Character optimization of one of the positions that directly affect the sister relationship between haptophytes (H) and the clade composed of stramenopiles, alveolates, and Rhizaria (SAR), in relation to the absence (A) or presence (B) of OTUs from intracellular endoparasites (including apicomplexans [AP]) and ciliates (CI) in the M 10∶16 data matrix set.** The optimization was performed using MacClade 4.08a in the fixed tree showing the clade composed of haptophytes and SAR. The arrow indicates the ancestral character elucidated below the SAR-H branch (bearing the clade composed of haptophytes and SAR). Note that the resolved ancestral characters differ between these two trees.
(TIF)

Figure S3 **Character optimization of one of the positions that directly affect the sister relationship between haptophytes (H) and the clade composed of stramenopiles, alveolates, and Rhizaria (SAR), in relation to the absence (A) or presence (B) of OTUs from intracellular endoparasites (including apicomplexans [AP]) and ciliates (CI) in the M 10∶16 data matrix set.** The optimization was performed using MacClade 4.08a in the fixed tree showing the clade composed of haptophytes and SAR. The arrow indicates the ancestral character determined below the SAR-H branch (bearing the clade composed of haptophytes and SAR). Note that the derived character (glycine) resolved at the SAR-H branch (A) disappeared in the lower tree (B).
(TIF)

Table S1 **Sequence data of five additional OTUs used for the present phylogenetic analyses (** **Figure 1** **and Figure S1).**
(DOC)
