# Supplementary figures and images for: A Case Study for Effects of Operational Taxonomic Units from Intracellular Endoparasites and Ciliates on the Eukaryotic Phylogeny: Phylogenetic Position of the Haptophyta in Analyses of Multiple Slowly Evolving Genes
Source: PLoS One. 2012 Nov 30;7(11):e50827. doi: 10.1371/journal.pone.0050827 (PMC3511332; doi:10.1371/journal.pone.0050827)

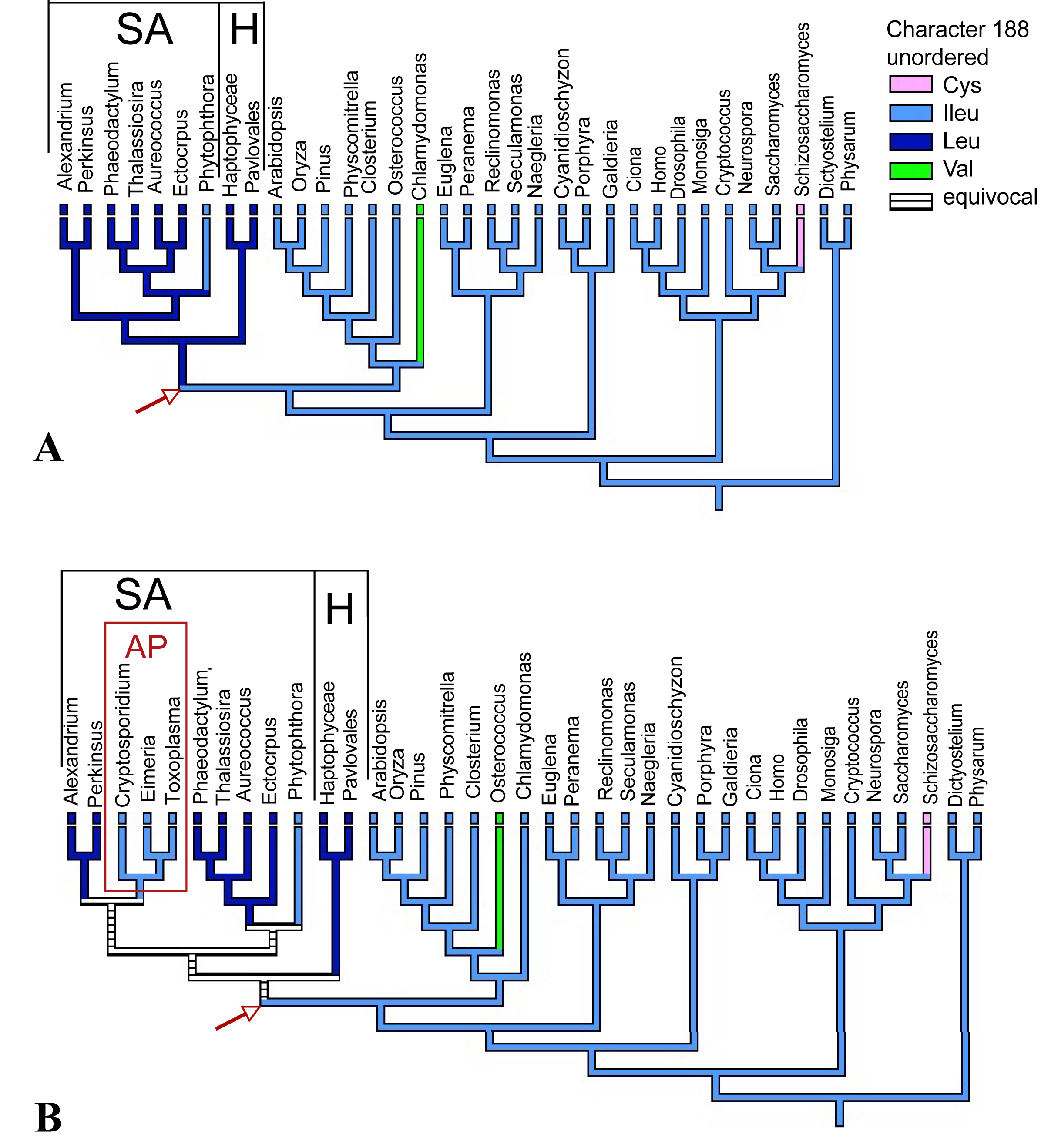

Supplement: Figure S1 — Character optimization of one of the amino acid positions that directly affected the sister relationship between haptophytes (H) and the clade composed of stramenopiles and alveolates (SA), in relation to the absence (A) or presence (B) of the intracellular endoparasite apicomplexan OTUs (AP) in the 6,048 aa data matrix set. Optimization was conducted using MacClade 4.08a in the fixed tree showing the clade composed of haptophytes and SA. The arrow indicates the ancestral character determined below the SA-H branch (bearing the clade composed of haptophytes and SA). Note that the derived character resolved at the SA-H branch (A) disappeared in the lower tree (B). (TIF) [file pone.0050827.s001.tif]

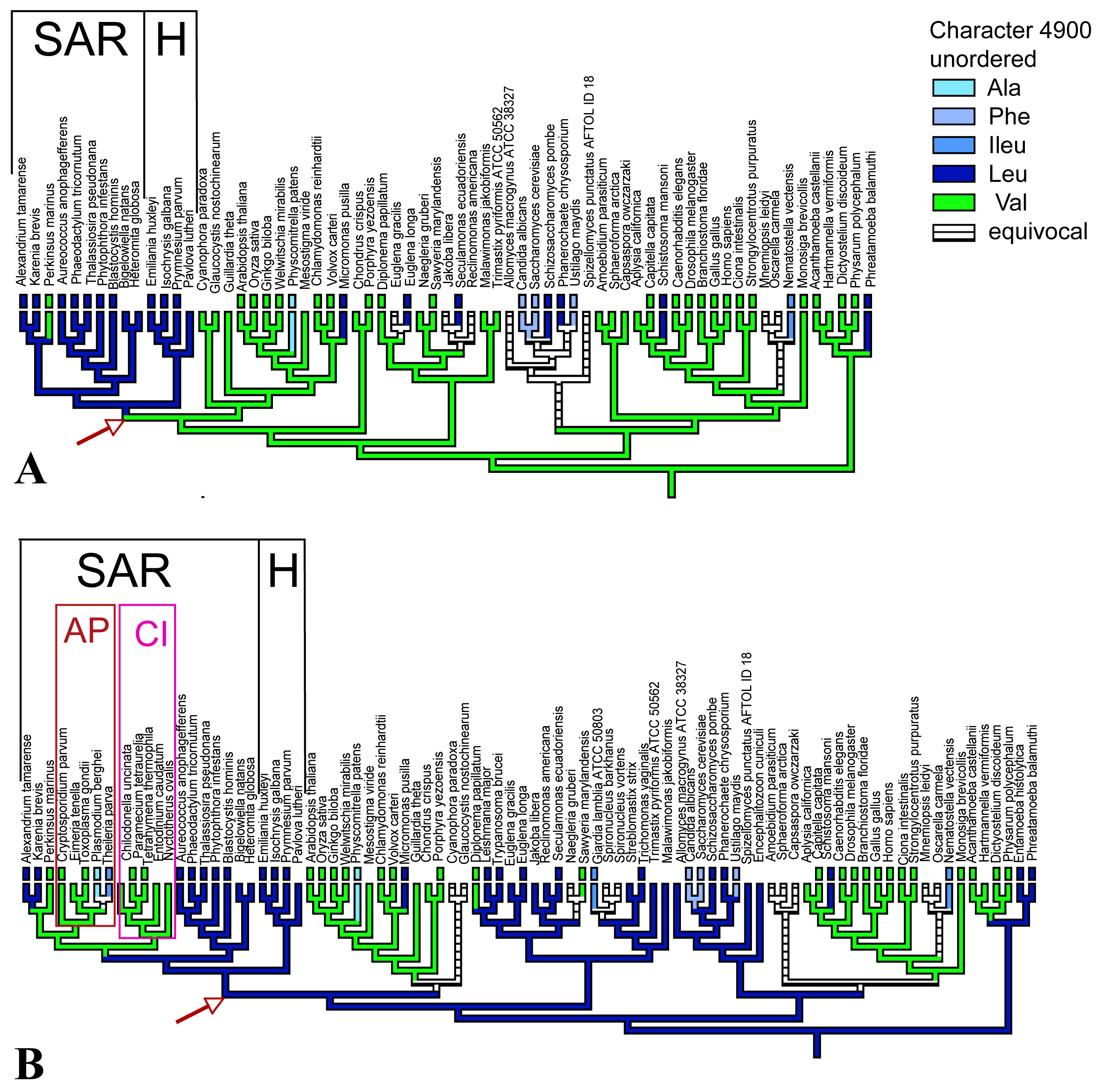

Supplement: Figure S2 — Character optimization of one of the positions that directly affect the sister relationship between haptophytes (H) and the clade composed of stramenopiles, alveolates, and Rhizaria (SAR), in relation to the absence (A) or presence (B) of OTUs from intracellular endoparasites (including apicomplexans [AP]) and ciliates (CI) in the M 10∶16 data matrix set. The optimization was performed using MacClade 4.08a in the fixed tree showing the clade composed of haptophytes and SAR. The arrow indicates the ancestral character elucidated below the SAR-H branch (bearing the clade composed of haptophytes and SAR). Note that the resolved ancestral characters differ between these two trees. (TIF) [file pone.0050827.s002.tif]

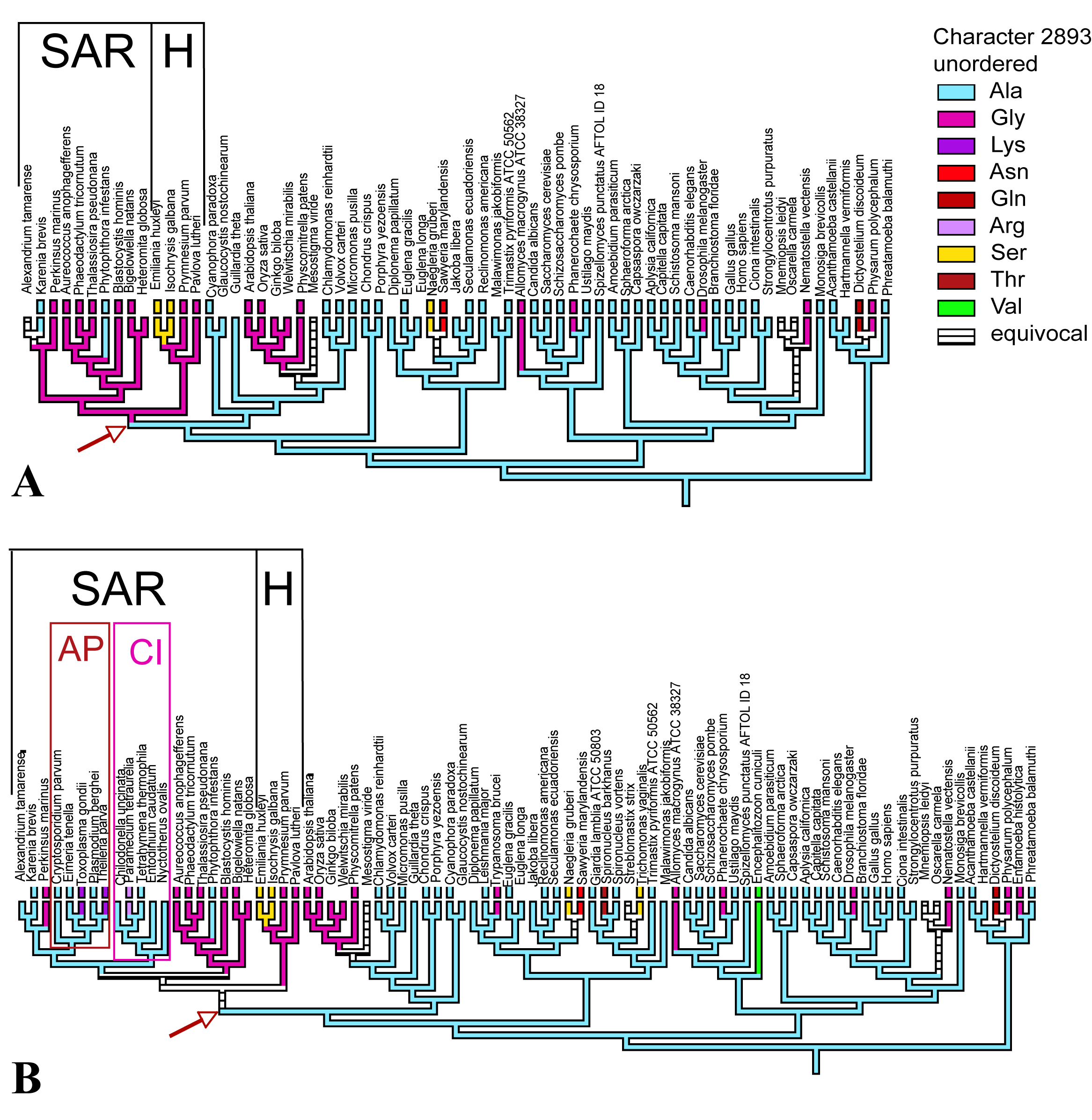

Supplement: Figure S3 — Character optimization of one of the positions that directly affect the sister relationship between haptophytes (H) and the clade composed of stramenopiles, alveolates, and Rhizaria (SAR), in relation to the absence (A) or presence (B) of OTUs from intracellular endoparasites (including apicomplexans [AP]) and ciliates (CI) in the M 10∶16 data matrix set. The optimization was performed using MacClade 4.08a in the fixed tree showing the clade composed of haptophytes and SAR. The arrow indicates the ancestral character determined below the SAR-H branch (bearing the clade composed of haptophytes and SAR). Note that the derived character (glycine) resolved at the SAR-H branch (A) disappeared in the lower tree (B). (TIF) [file pone.0050827.s003.tif]
