# Supplementary material for: A Case Study for Effects of Operational Taxonomic Units from Intracellular Endoparasites and Ciliates on the Eukaryotic Phylogeny: Phylogenetic Position of the Haptophyta in Analyses of Multiple Slowly Evolving Genes
Source: PLoS One. 2012 Nov 30;7(11):e50827. doi: 10.1371/journal.pone.0050827 (PMC3511332; doi:10.1371/journal.pone.0050827)
Supplement: Table S1 — Sequence data of five additional OTUs used for the present phylogenetic analyses ( Figure 1 and Figure S1). (DOC) [file pone.0050827.s004.doc]

Table S1. Sequence data of five additional OTUs used for the present phylogenetic analyses (figs. 1 and S1).

| Taxon | actin-A | EF-1alpha | grc5 | h4 | hsp70-C |
| --- | --- | --- | --- | --- | --- |
| Ectocarpus | CBJ30601 | CBJ32893 | CBN75612 | CBJ33198 | CBJ32839 |
| *Aureococcus* | jgi|Auran1|60226 | jgi|Auran1|36932 | jgi|Auran1|27934 | jgi|Auran1|60370 | jgi|Auran1|36205 |
| *Euglena* | AF057161 | ESTs | Q39724 | No sequence | ELL00002598 |
| *Peranema* | AB757690-1 | AB757692-4 | No sequence | No sequence | AB757695-8 |
| *Seculamonas* | EC817126, EC816983, EC814252 | EC817322, EC817068, EC816308 | EC814989 | EC684256 (*Jakoba bahamiensis*) | EC813968, EC812673 |

*For *Chlamydomonas* EF-1alpha, *Coccomyxa* sequence (>jgi|Coc_C169_1|27513|estExt_Genewise1Plus.C_30283) was used (figs. 1 and S1).

Table S1. Continued

| Taxon | hsp70-E | nsf1-I | nsf1-M | rpl2 | rpl27 |
| --- | --- | --- | --- | --- | --- |
| Ectocarpus | CBJ48460 | FP253360, CBJ33290 | CBJ33278 | CBJ28111 | CBJ26345 |
| *Aureococcus* | JGI EST >4211177:325 | jgi|Auran1|31970 | JGI_CATU8227.fwd,  JGI_CATU8227.fwd,  JGI_CATU36198.fwd,  JGI_CATU36198.fwd | jgi|Auran1|54227 | JGI_CATU30943.fwd,  JGI_CATU30943.fwd |
| *Euglena* | ELL00001708 | ELL00008100 | ELL00001460 | ELL00003070 | EC674489 |
| *Peranema* | AB757702-5 | No sequence | No sequence | AB757683 | No sequence |
| *Seculamonas* | EC817218 | EC815197 (*Seculamonas*),  EC685219  (*Jakoba bahamiensis*) | EC687659,  EC686517  (*Jakoba bahamiensis*) | EC816949 | EC693052  (*Jakoba libera*) |

Table S1. Continued.

| Taxon | rpl3 | rpl42 | rps2 | rps14 | rps17 |
| --- | --- | --- | --- | --- | --- |
| Ectocarpus | CBJ28620 | CBJ32132 | CBJ32526 | CBJ26566 | CBJ26577 |
| *Aureococcus* | jgi|Auran1|36266 | jgi|Auran1|30290 | jgi|Auran1|31983 | jgi|Auran1|70003 | jgi|Auran1|36910 |
| *Euglena* | ELL00000305 | ELL00000177 | EC673564 | EC670868 | ELL00000074 |
| *Peranema* | AB757706 | No sequence | No sequence | No sequence | AB757699 |
| *Seculamonas* | EC816642, EC816738 | EC813094 | EC815271 | EC815408 | EC813305 |

Table S1. Continued.

| Taxon | rps23 | rps5 | vatb | hps90 |
| --- | --- | --- | --- | --- |
| Ectocarpu | CBJ31696 | CBJ48704 | CBN75478, FP295621 | CBJ29197 |
| *Aureococcus* | jgi|Auran1|28783 | jgi|Auran1|59875 | jgi|Auran1|63073 | jgi|Auran1|31909 |
| *Euglena* | ELL00007900 | EC672004 | ELL00001638,  ELL00007418 | AAQ24862 |
| *Peranema* | AB757700 | AB757701 | AB757707 | AB757684-9 |
| *Seculamonas* | EC815364 | EC817165 | EC816776, EC815173, EC816777 | EC813030, EC813480,  EC815655, EC817244 |
|  |  |  |  |  |

Table S1. Continued.

| Taxon | Alpha-tubulin | Beta-tubulin |  |
| --- | --- | --- | --- |
| Ectocarpus | CBN77373 | CBN79446 |  |
| *Aureococcus* | jgi|Auran1|60342 | jgi-Auran1|29718 |  |
| *Euglena* | Z22877 | X15797 |  |
| *Peranema* | AB757677-9 | AB757680-2 |  |
| *Seculamonas* | EC817319, EC815522, EC814925 | EC816637, EC816359 |  |
